# Supplementary figures and images for: Spontaneous urinary bladder regeneration after subtotal cystectomy increases YAP/WWTR1 signaling and downstream BDNF expression: Implications for smooth muscle injury responses
Source: PLoS One. 2023 Jul 26;18(7):e0287205. doi: 10.1371/journal.pone.0287205 (PMC10370683; doi:10.1371/journal.pone.0287205)

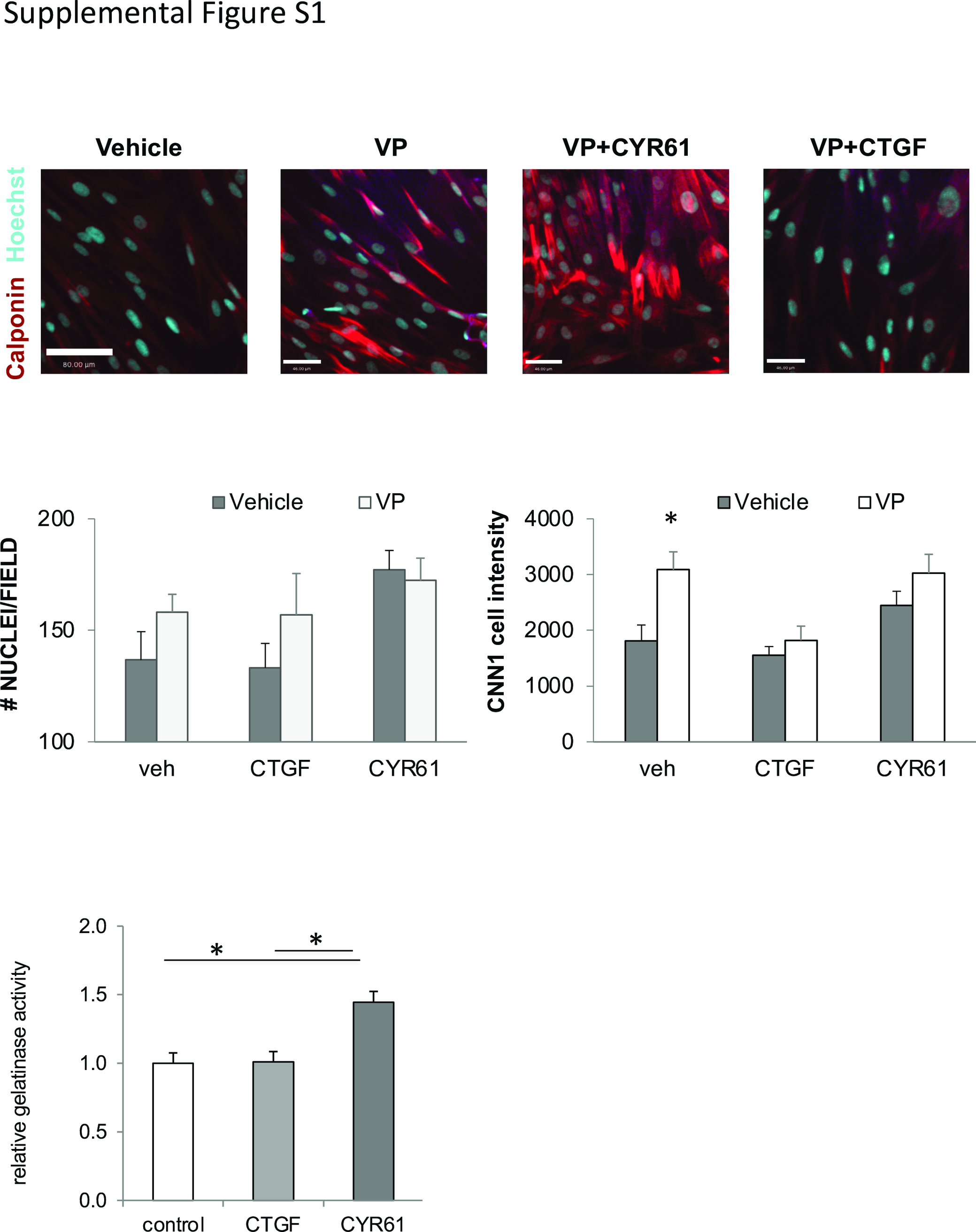

Supplement: S1 Fig — SMC on denatured collagen, were treated with vehicle or the YAP/WWTR1 inhibitor (Verteporfin, 0.1 μM). Addition of CTGF was able to decrease VP-induced differentiation (p<0.05, vs. VP alone), in contrast to CYR61, which did not decrease the effect of VP. (TIF) [file pone.0287205.s001.tif]

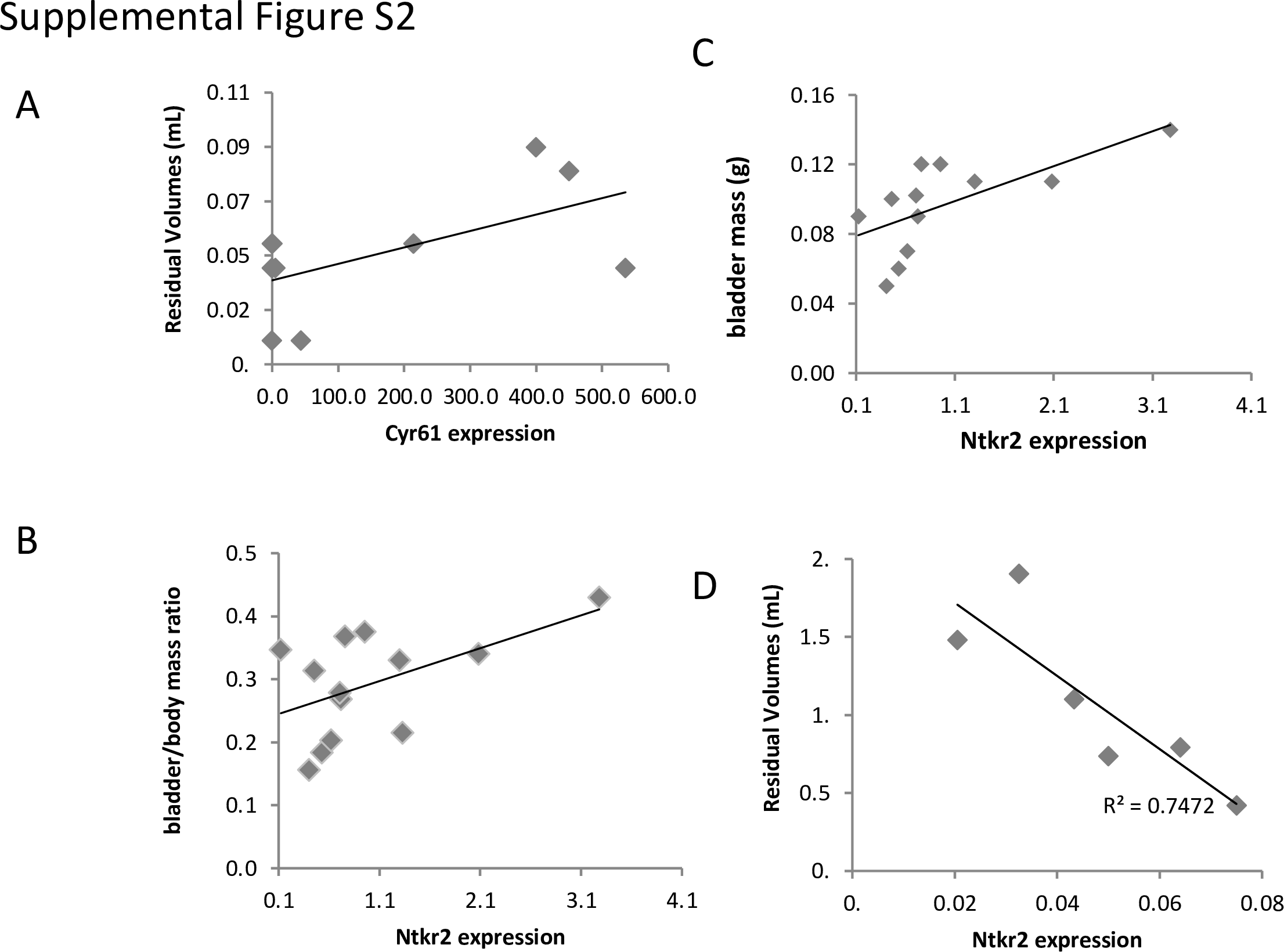

Supplement: S2 Fig — Pearson’s correlations were performed with delta or linear values from QPCR for CYR61 (A) and NTRK2 with or bladder/body mass ratios (B), bladder mass (C) and residual volumes (D). (TIF) [file pone.0287205.s002.tif]

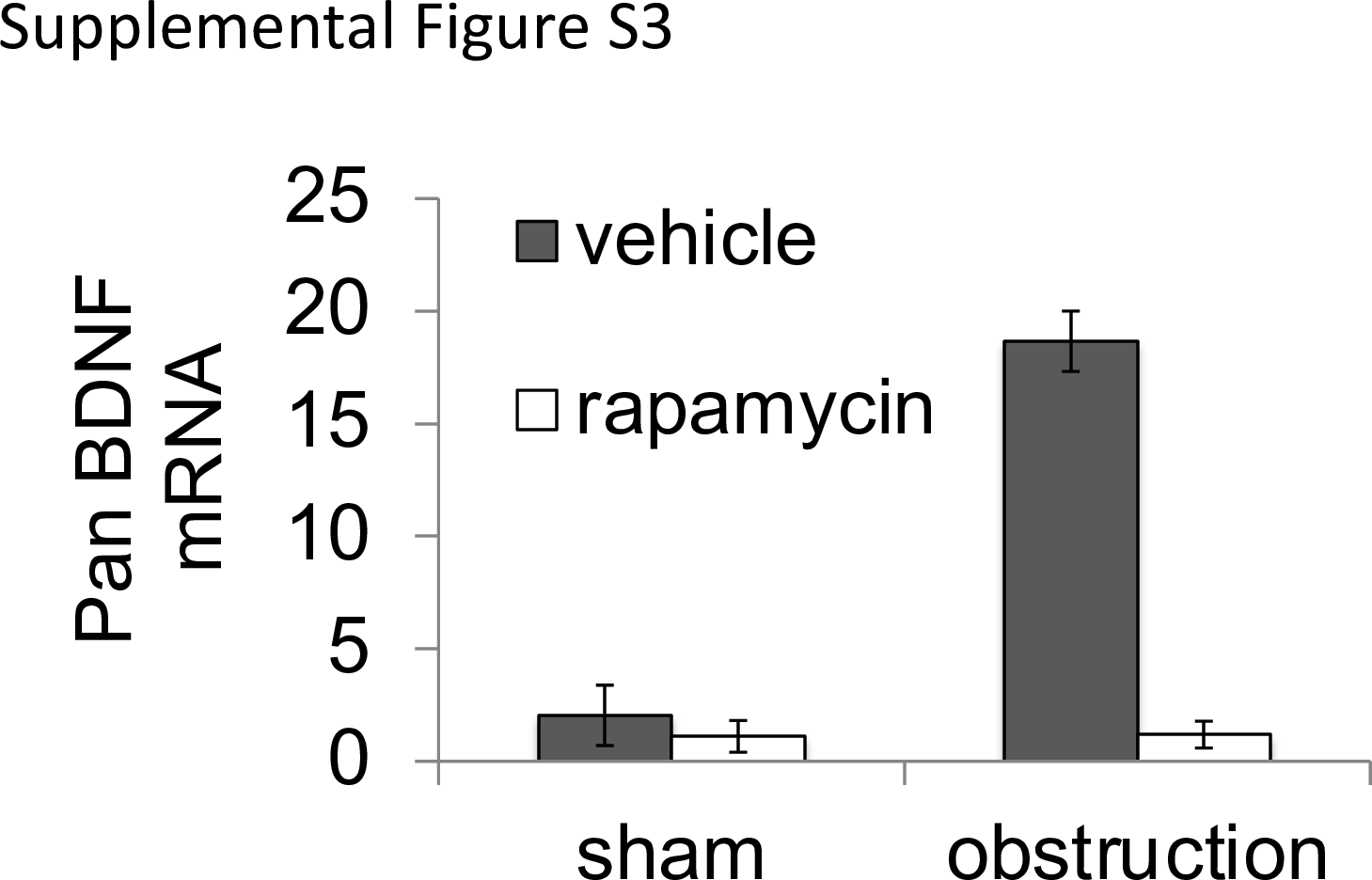

Supplement: S3 Fig — Expression of Pan BDNF is significantly increased in obstructed bladders in comparison to sham, p<0.01. However, Pan BDNF expression is similar between both sham and obstruction bladders upon treatment with rapamycin, an mTOR inhibitor. This animal study was previous published(Schröder et al, 2013, ref 1). Archival cDNA from this study was amplified with panBDNF primers and compared to reference gene results by ddct methods. 1. Schröder A, Kirwan TP, Jiang JX, Aitken KJ, Bägli DJ. Rapamycin Attenuates Bladder Hypertrophy During Long-Term Outlet Obstruction In Vivo: Tissue, Matrix and Mechanistic Insights. J Urology. 2013 Jun;189(6):2377–84. (TIF) [file pone.0287205.s003.tif]
